# Bayesian Approaches for Confirmatory Trials in Rare Diseases: Opportunities and Challenges

**DOI:** 10.3390/ijerph18031022

**Published:** 2021-01-24

**Authors:** Moreno Ursino, Nigel Stallard

**Affiliations:** 1 Inserm, Centre de Recherche des Cordeliers, Sorbonne Université, USPC, Université de Paris, F-75006 Paris, France; 2 F-CRIN PARTNERS Platform, AP-HP, Université de Paris, F-75010 Paris, France; 3 Statistics and Epidemiology, Division of Health Sciences, Warwick Medical School, University of Warwick, Coventry CV4 7AL, UK; n.stallard@warwick.ac.uk

**Keywords:** Bayesian, rare disease, prior distribution, meta-analysis, sample size

## Abstract

The aim of this narrative review is to introduce the reader to Bayesian methods that, in our opinion, appear to be the most important in the context of rare diseases. A disease is defined as rare depending on the prevalence of the affected patients in the considered population, for example, about 1 in 1500 people in U.S.; about 1 in 2500 people in Japan; and fewer than 1 in 2000 people in Europe. There are between 6000 and 8000 rare diseases and the main issue in drug development is linked to the challenge of achieving robust evidence from clinical trials in small populations. A better use of all available information can help the development process and Bayesian statistics can provide a solid framework at the design stage, during the conduct of the trial, and at the analysis stage. The focus of this manuscript is to provide a review of Bayesian methods for sample size computation or reassessment during phase II or phase III trial, for response adaptive randomization and of for meta-analysis in rare disease. Challenges regarding prior distribution choice, computational burden and dissemination are also discussed.

## 1. Introduction

A disease is defined as rare depending on the prevalence of the affected patients in the considered population. In the United States, a disease is rare if it affects fewer than 200,000 people in the U.S. [1] (or about 1 in 1500 people); in Japan, if it affects fewer than 50,000 patients in Japan (or about 1 in 2500 people); and in the European Union if the prevalence is no more than 5 per 10,000 (that is, fewer than 1 in 2000 people), but the definition excludes diseases that are not also life-threatening, chronically debilitating, or inadequately treated [2]. There are between 6000 and 8000 rare diseases [3], 71.9% of which are genetic and 69.9% which exclusively affect paediatric populations, and it is estimated that the global population prevalence of rare diseases is of 3.5–5.9%, which implies that 263–446 million persons are affected at any stage in their life [4]. The usual level of rigorous clinical trial evaluation of treatments is required in rare diseases just as much as in more common ones. Although in some cases, particularly in phase II trials, single-arm trials might be considered (see, for example, Grayling et al. [5]), randomized controlled trials are to be preferred when this is possible. For example, the European regulatory guidance [2] affirms that “patients with [rare] conditions deserve the same quality, safety and efficacy in medicinal products as other patients; orphan medicinal products should therefore be submitted to the normal evaluation process"; this is also in agreement with U.S. guidance [6].

The main issue in drug development for rare diseases is linked to the challenge of achieving robust evidence from clinical trials in small populations when trial sample sizes are necessarily limited [7]. Even if for some rare diseases the population size is relatively large (for instance, Friedreich Ataxia in the EU) [8], the majority of rare diseases are less frequent [9]. Small population clinical trials have been the focus of much methodological research activity in the last two decades. From a regulatory perspective, the European Medicines Agency (EMA) described a methodological framework, summarizing several possible approaches, in the guidance “Guideline on Clinical Trials in Small Populations” [10] and the Food and Drug Administration (FDA) in the draft guidance on rare disease [6]. The European Union’s Seventh Framework Programme for Research, Technological development and Demonstration (EU FP7), acknowledging the need for additional methodological research work, funded three projects in 2013; the Integrated Design and Analysis of Small Populations Group Trials (IDeAl) project (www.ideal.rwth-aachen.de), the Innovative Methodology for Small Populations Research (InSPiRe) project (www.warwick.ac.uk/inspire), and the Advances in Small Trials Design for Regulatory Innovation and Excellence (Asterix) project (www.asterix-fp7.eu) [8,11,12,13].

The drug development process involves on-going learning as data are observed through the series of clinical trials, and, above all in rare diseases, there is a considerable effort to optimize this learning process [14,15]. A better use of all available information can help the process and Bayesian statistics provides an opportunity to do this in a formal way (at the design stage, during the conduct of the trial, and at the analysis stage) [16,17]. Like drug development, the Bayesian approach can be seen as an on-going learning process: it starts with a prior belief (quantified as a prior distribution for the unknown model parameters), which is then updated with the new evidence (likelihood data from the new trial/experiment) to yield a posterior belief (expressed as a posterior probability distribution for the unknown model parameters). In this way, Bayesian statistics provides a mathematical method for calculating the predictive probabilities of future events, given the actual trial and the knowledge from prior trials. Moreover, a formal Bayesian analysis can incorporate different utilities or prior beliefs coming from different stakeholders and quantify how these could impact potential decision-making.

Bayesian methods and designs are well established and mostly accepted, by both clinicians and regulatory agencies, in early phase clinical trials. Due to the greater flexibility, in both design and analysis, of the Bayesian paradigm with respect to the frequentist one and since type I and II errors do not have to be controlled at this stage, Bayesian adaptive designs are mostly chosen for these stages [18]. As early phase trials in all diseases use small sample sizes, designs, specifically developed for rare diseases are unnecessary. Thus, in this manuscript we will focus on novel Bayesian approaches firstly developed for confirmatory/randomized trials in the rare disease setting, where more conventional approaches may be unfeasible.

The aim of this narrative review is to show to the reader Bayesian methods that, in our opinion, appear to be the most important in the context of rare diseases. Its purpose is not to present a comprehensive compendium of Bayesian statistics in rare diseases, but to give a starting point for the reader on some uses of novel Bayesian methods in this field. All methods are presented in a general way, without mathematical formalism, so that a reader who already have a basis of Bayesian statistics can understand the general idea, along with the corresponding principal(s) reference(s), in such a way the reader can find the details for methods they wish to explore in more detail. In the following sections, three specific topics dealing with the application of a Bayesian design are introduced. The first topic, in Section 2, regards methods for sample size computation or reassessment during randomized phase II or phase III trial. The second topic, a recent method proposed for response adaptive randomization, is presented in Section 3. Section 4 presents the fourth topic of Bayesian meta-analysis methods developed for evidence combination in rare diseases. Finally, we discuss frequent challenges faced when choosing a Bayesian design; in particular, the priors distributions choice, which include the issue of the quantity of information and commensurability between prior information and actual data; the computational burden required; and dissemination issues.

## 2. Sample Size Determination/Re-Estimation

Usual sample size determination approach focus on frequentist properties, that is, type I error and study power. However, in a rare disease setting, accruing the number of patients required to perform a fully-powered significance test after the trial may be infeasible. Increasing the allowed type I error can be a solution to reduce the required sample size. However, as recalled in the introduction, this practice is not generally supported by regulatory agencies. As an alternative, some authors have proposed the use of a Bayesian decision-theoretic framework [19]. A Bayesian decision-theoretic approach can be applied when we would like a treatment recommendation not based on type I error but on maximizing an expected gain for the total population. According this approach, we can compare the costs of clinical trial evaluation with the potential benefits to current and future patients, assessing how the cost-benefit balance differs between large and small patient populations when, in the latter, patients recruited to a clinical trial could be a substantial proportion of the population. The design of the study, including the sample size, can then be chosen on the basis of the expected gain, with the sample size that maximizes the expected gain chosen for the clinical study. Here the concept of a “gain” is interpreted very broadly and can be defined from the patient, sponsor, regulatory, public health, or society perspective, or from a combined perspective. Stallard et al. [20] have shown that for a wide range of distributions, including those for continuous, binary or count responses, and gain function forms, the optimal trial sample size is proportional to the square root of the population size, with the constant of proportionality depending on the gain function form and prior distribution of the parameters of the distribution of the data. A smaller sample size may thus be appropriate for a trial in a rare disease than in a more common one.

Bayesian statistics can also be adopted to overcome some challenges in the calculation of sample sizes from the frequentist perspective. For example, for normally distributed outcomes, values for variances need to be specified, but, especially in the case of small populations, may be based on very little information, for example, that from only one very small pilot study. When using a Bayesian approach, the aggregation of prior information on the variance with newly collected data is more formalized. Brakenhoff et al. [21] proposed a framework incorporating the employment of power priors in order for operational characteristics to be controlled in case of prior-new data conflict.

Bayesian group sequential designs could also be used to provide interim stopping criteria, based on efficacy and/or futility. Even if the frequentist operating characteristics of these designs are usually checked, they are not designed to optimize them. A practical guide for their implementation and reference for software can be found in Gsponer et al. [22].

## 3. Response Adaptive Randomization

While randomization is the established method for obtaining scientifically valid treatment comparisons in clinical trials, as the trial progresses, increasing evidence may suggest that one study group is responding or doing much better than another. As a consequence, novel randomization methods, such as response adaptive randomization [23] (RAR), have been proposed to address this ethical question continuously updating assignment probabilities based on response of the different groups to their respective treatments so as to allocate more patients to better-performing treatments. Both, frequentist and Bayesian approach can be applied, however, the latter one has gained more popularity due to its flexibility [24,25]. In the same manner as the previous decision theoretic idea, this approach could be considered in rare disease setting, where future patients in the general population is limited, to balance the benefits to current trial patients and future ones. Nonetheless, standard adaptive randomization may lead to estimation bias [26], with the potential for the trial to reach an erroneous conclusion. Therefore, novel and calibrated RAR approaches should be preferred. The small sample sizes in trials in rare diseases may also mean that it is possible to calibrate RAR methods in a way that would be infeasible in larger trials.

A recent paper suggests a novel randomized response-adaptive design specifically developed for a rare disease trial [27]. It uses the framework of finite-horizon Markov decision processes and dynamic programming (DP) to recruit more patients to the more beneficial arms while guaranteeing a minimum sample size to each treatment arm. The authors show that the design has good operating characteristics, in term of (i) the percentage of patients allocated to the superior arm, which is much higher than in the traditional fixed randomized design; (ii) the power, which is higher than optimal DP; and (iii) bias and mean square error of the treatment effect estimator, which are small.

## 4. Meta-Analysis

Meta-analyses are used to combine evidence from multiple studies. Differences in study characteristics, such as trial design and study populations, can bring to heterogeneous treatment effects and these must be accounted for in the meta-analysis formulation. To deal with the between-trial heterogeneity, random-effects meta-analysis has become the gold standard, and the most used method is the normal–normal hierarchical model (NNHM) [28]. In a rare disease, the limited number of trials and their small sample size may impact the validity of usual frequentist meta-analysis methods. A Bayesian approach offers another way to perform random-effect meta-analyses within the NNHM framework. One of the advantages is that the solution remains coherent for small numbers of studies, although careful prior specification is required. Friede et al. [29] showed that, when doing meta-analysis with only two studies, Bayesian random-effects meta-analyses with priors covering plausible heterogeneity values offer a good compromise. They compared the Bayesian method to the NNHM, to the Hartung-Knapp-Sidik-Jonkman method (HKSJ) and to the modified Knapp-Hartung method (mKH). On one hand, the coverage of the standard method, based on normal quantiles, was unsatisfactory; on the other hand, very large (therefore uncertain) confidence intervals resulted from the HKSJ and mKH. An acceptable trade-off between these two extremes was achieved, in general, by Bayesian intervals that showed suitable characteristics. Usually, the Bayesian approach is computationally more demanding. However, optimized free software are available, such as the bayesmeta R package, which uses a general semi-analytical approach to solve the meta-analysis problem via the DIRECT approach [30] and provides an efficient and user-friendly interface to Bayesian random-effects meta-analysis [31].

When dealing with binary outcomes, the binomial-normal hierarchical model is usually preferred to the NNHM, which then relies on asymptotic approximations. A challenge in this setting in rare diseases is that we could face the probability to have no events due to the small sample sizes. Frequentist approaches are known to induce bias and to result in improper interval estimation of the overall treatment effect in a meta-analysis with zero events [13]. On the other hand, Bayesian models are known for being sensitive to the choice of heterogeneity prior distributions in sparse settings, therefore, the need to identify priors with robust properties is crucial. Pateras et al. [32] proposed a general way to set prior distributions. Via simulations, they showed that a uniform heterogeneity prior, bounded between -10 and 10, on the log heterogeneity parameter scale shows appropriate 95% coverage and induces relatively acceptable under/over estimation of both the overall treatment effect and heterogeneity, across a wide range of heterogeneity levels.

The Bayesian meta-analysis approach also allows implementation of a number of more advanced analysis strategies. A series of studies may be used to inform the analysis when the focus is not on an overall synthesis, but rather on a particular study that is to be viewed in the light of previously accumulated evidence. For example, Wandel et al. [33] used a Bayesian meta-analytic approach to inform a phase III study with phase II data. They investigated the use of shrinkage estimates to support data from a single trial in the light of external information. The method allows quantifying and discounting the phase II data through the predictive distribution relevant for phase III. Bayesian meta-analysis approach can also be adapted to incorporate external information from historical controls [34] or borrow information from other arms in a randomized control trial, for example, in a basket design [35,36]. Such approaches could prove very valuable in the setting of rare diseases where trials are necessarily small.

## 5. Challenges

As stated above, the Bayesian approach can be more flexible than the frequentist counterpart. However, the flexibility comes along with a number of possible challenges. Even if Bayesian methods can bring substantial benefits, their validity and effectiveness require expertise and care. In the following, we will describe some points that should be addressed when planning a Bayesian analysis.

### 5.1. Prior Distributions Choices

In Bayesian statistics, external information can be easily incorporated into the prior distributions. An informative prior distribution for the unknown parameters could be determined through elicitation of expert knowledge, from data from other trials or from a search of the literature to identify results obtained in trials of similar drugs, or the same drug in a different population, via the so called “extrapolation". Extrapolation approaches are well known in paediatrics, where the proper dosage for children is estimated starting from adults’ data, and in bridging studies, where the drug is tested in a new geographical population, for example, in Asian, given the results in a previous one, for example, Caucasian. This concept can be translated in rare disease, since rare diseases prevalence may vary by continent (i.e., IgA nephropathy is rather rare in the EU but more frequent in Asia and Africa) and we can be to adopt proofs of efficacy from the larger populations to the smaller one [8].

However the prior distribution is obtained, the use of an informative prior to make inferences about medical treatments based on small sample size trials remains inherently controversial, however. Choice of a prior distribution must therefore be done carefully, since the use of informative priors may be seen as introducing bias into posterior inferences and inflating type I error rates. This is a general problem common in many different fields, and several authors have addressed the issue of eliciting experts’ opinions, building priors upon the elicited values, and performing Bayesian analyses using the resulting priors. See O’Hagan et al. [37] for a complete review. The elicitation needs to be made as meticulous and objective as possible to catch expert expertise. One way is to follow a recognized protocol that is designed to address and minimize the cognitive biases [38]. In the following, we summarize two approaches that have already been used in the context of rare disease.

The first approach describes how to obtain a consensus between experts. This research was motivated by the design of the MYPAN trial, a multicentre RCT comparing mycophenolate mofetil (MMF) with cyclophosphamide (CYC) for the treatment of polyarteritis nodosa, a rare and serious inflammatory blood vessel disease in children [39]. The authors proposed to add priors on the probability of success of one arm and on the log-odds ratio of the probabilities. Then, a behavioral aggregation process, by which experts interact to reach a mutually agreeable consensus through constructive discussions, was chosen for systematic elicitation from clinicians of their beliefs concerning treatment efficacy. In particular, experts’ individual prior beliefs were obtained at the beginning of the process; then, the full group was asked to reach a consensus. The results are then used to establish Bayesian priors for unknown model parameters and the authors have also considered the possibility of considering results from related trials. A similar strategy was used in a trial of adalimumab versus pamidronate for children with CNO/CRMO [40].

The second approach focuses on reflecting, when eliciting experts’ opinions, how these depend on differences in experience, training and medical practice [41]. Motivating by a 70-patient randomized trial to compare two treatments (the same described in the first approach) for idiopathic nephrotic syndrome in children (NCT 01092962), the authors proposed a Bayesian methodology for constructing a bivariate parametric prior starting from elicited graphical information. The method involves four steps: (i) each physician builds manually two histograms, one for each treatment parameter using the “bins-and-chips” graphical method of Johnson et al. [42]; (ii) then, for each physician and each treatment parameter, a marginal prior, characterized by location and precision hyperparameters, is fitted to the elicited histogram; (iii) a bivariate prior is built by averaging the marginals over a latent bivariate distribution; (iv) finally, an overall prior is obtained as a mixture of the individual physicians’ priors. The approach also suggests a framework for performing a sensitivity analysis of posterior inferences to prior location and precision.

Incorporating external information, whatever the source type (other trial, experts’ elicitation, etc.), has to be done properly, as the information can be in conflict with the actual data or the amount of information can overwhelm trial data. Several methods that allow prior information to be incorporated if it is in accordance with the trial data and otherwise to be down-weighted have been proposed [34,43,44]. Moreover, the effective sample size allows to quantify the information in the prior to be specified in term of the number of hypothetical patients used to build the prior [45]. Different prior building approaches may be used for different parameters; for example, historical control can be included via a power prior approach and experts’ opinion can be used for the new treatment effect. An adaptation of the power prior approach, that is useful particularly for borrowing evidence from a single historical study, was proposed in rare disease setting [46]. Borrowing information from a historical trial is often related the type I error inflation. By determining the amount of similarity between the new and historical data, this method uses predictive probabilities and is parameterized in order to control the type I error.

### 5.2. Computational Burden

Estimation of posterior distributions can be challenging when prior distributions do not have simple conjugate forms. Specific Markov chain Monte Carlo algorithms, such as the Gibbs sampling or the Hamiltonian Monte Carlo, can be used to obtain an approximation of posteriors. Even if freely available software and the increasing computational power of computers may help the Bayesian implementation, writing, coding and testing the models usually requires a bigger effort than choosing a frequentist approach. In general, the more complex the model or the prior distribution, the longer the computational time to obtain the result. Validation of the method via simulation is one of the common way used in Bayesian setting. Simulating several possible scenarios can allow the user to calibrate model parameters (i.e., the quantity of information in the prior distribution) to obtain desired operational characteristics, such as the type I error control or the power. Choosing a fast and reliable approximation method is, however, crucial when simulations are required.

### 5.3. Dissemination

Even if the Bayesian approach has been shown to capture the thinking behavior of clinicians [41], Bayesian methods and results are sometimes still viewed with suspicion by clinicians and traditional statisticians. The influence of the prior distribution may be considered disturbing and the lack of p-values can give the feeling that regulatory agencies will not consider the results obtained. In effect, Bayesian methodologies are usually less discussed in public regulatory guidelines than the frequentist counterpart. However, the FDA Guidance for the Use of Bayesian Statistics in Medical Device Clinical Trials [47] shows the regulatory agencies efforts in this direction. The guidance gives several Bayesian insights that could be used in general, not only in medical device field. Several sections explain how to well plan a Bayesian clinical trial, what to consider when choosing prior distributions, and how to analyze the data. Then, in the technical details sections, the FDA points out the importance of simulations to obtain operating characteristics of the planned design, to assess the type one error rate, power, etc. While also other recommendations for Bayesian analyses have been developed [17,48,49], they were primarily addressed to researchers, not to readers unfamiliar with Bayesian approaches [50]. Therefore, efforts are needed to well explain the Bayesian philosophy to non-statisticians. An example is given in Ferreira et al. [51] and Ferreira et al. [50], where the authors help clinicians interpretation of Bayesian clinical trial though a side-by-side comparison with the frequentist approach. On one hand, they teach how to transfer frequentist ideas, such as the p-values or hypotheses testing, to the Bayesian framework, such as posterior probabilities and Bayes factor, and, on the other hand, they give insights on what to check when reading a Bayesian report.

## 6. Conclusions

The aim of this article has been to review the use of Bayesian methods in confirmatory trials in rare diseases, though many of the approaches described could also be applied in clinical trials in other more common disease areas.

The formal Bayesian approach permits the incorporation of accumulating information into the analysis of the actual trial and, therefore, the updating of belief. This feature is extremely attractive in rare disease setting, where usually sample sizes and the opportunities to performs clinical trials are limited. Incorporation of previous information should be strongly considered and the Bayesian approach, with its flexibility, could be seen as a future gold standard in this field. As shown in the manuscript, the accumulated information can be used in the prior distribution settings, in sample size optimization and/or in randomization. Depending on the trial, where and when using this kind of information is used has to be carefully chosen and simulations are strongly suggested to evaluate method performances.

## Abbreviations

The following abbreviations are used in this manuscript:

CYC	cyclophosphamide
CNO/CRMO	chronic recurrent multifocal osteomyelitis
DP	dynamic programming
EMA	European Medicines Agency
FDA	Food and Drug Administration
HKSJ	Hartung-Knapp-Sidik-Jonkman method
mKH	modified Knapp-Hartung method
MMF	mycophenolate Mofetil
NNHM	normal-normal hierarchical model
RAR	response adaptive randomization
